# Gastrointestinal nematode infection during pregnancy and lactation enhances spatial reference memory and reduces indicators of anxiety-like behaviour in uninfected adult female mouse offspring

**DOI:** 10.1017/S0031182024000696

**Published:** 2024-06

**Authors:** Sophia Noel, Ryan LaFrancois, Marilyn E. Scott

**Affiliations:** Institute of Parasitology, McGill University (Macdonald Campus), Quebec H9X 3V9, Canada

**Keywords:** anxiety-like behaviour, Barnes maze test, gastrointestinal nematode, *Heligmosomoides bakeri*, maternal infection, mouse model, offspring behaviour, spatial learning and memory

## Abstract

Maternal bacterial and viral infections that induce neuroinflammation in the developing brain are associated with impaired cognitive function and increased anxiety in the offspring. In contrast, maternal infection with the immunoregulatory murine gastrointestinal (GI) nematode, *Heligmosomoides bakeri*, appears to benefit neurodevelopment as juvenile 2- and 3-week-old male and female offspring had enhanced spatial memory, which may be due to a Th2/Treg biased neuroimmune environment. Here, the impact of maternal *H. bakeri* infection during pregnancy and lactation on the spatial and anxiety-like behaviours of adult, 3-month-old uninfected male and female offspring was explored for the first time. It was observed that adult female offspring of *H. bakeri*-infected dams had enhanced spatial reference memory and reduced anxiety-like behaviour compared to females of uninfected dams. These effects were not observed in adult male offspring. Thus, the positive influence of a maternal GI nematode infection on spatial memory of juvenile offspring persists in adult female offspring.

## Graphical abstract

**Figure:**
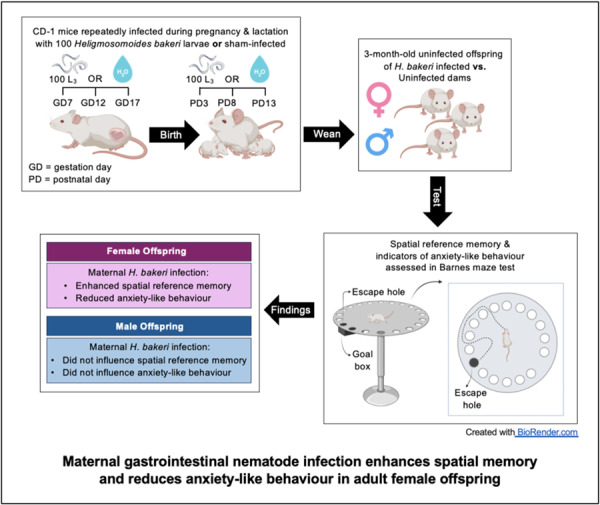

## Introduction

Brain development is a highly plastic process that, in rodents, starts *in utero* and continues postnatally until 3 months of age when brain maturation is completed (Hammelrath *et al*., 2016). During this vulnerable period, environmental stimuli such as maternal physical exercise favour brain development (Robinson and Bucci, 2014; Gomes da Silva *et al*., 2016), whereas exposure to bacterial and viral infections or toxins during pregnancy impairs neurodevelopment (Boksa, 2010; Wilhelm and Guizzetti, 2015; Bergdolt and Dunaevsky, 2019; Beversdorf *et al*., 2019). There has been a large effort over the past 2 decades to understand the link between maternal exposure to these pathogens and the risk of neurological disorders in offspring with a developmental origin, including autism spectrum disorder and schizophrenia (Boksa, 2010; Bergdolt and Dunaevsky, 2019). With the help of rodent models, it is now understood that the maternal immune response, not a specific pathogen, is a risk factor for neurodevelopmental disorders (Bergdolt and Dunaevsky, 2019). Viral or bacterial pathogens or their mimics [polyinosinic–polycytidylic acid (Poly I:C) or lipopolysaccharide (LPS) respectively] induce a strong pro-inflammatory immune response in the mother, which extends to the offspring, resulting in an altered immune profile in the developing brain which ultimately leads to irreversible neurodevelopmental defects and the emergence of behavioural abnormalities and cognitive impairments (Boksa, 2010; Bergdolt and Dunaevsky, 2019). Interestingly, however, it was observed that maternal exposure to the immunoregulatory gastrointestinal (GI) nematode, *Heligmosomoides bakeri* (also referred to as *Heligmosomoides polygyrus* and previously known as *Nematospiroides dubius*), may actually benefit, not harm, at least some aspects of brain development of the offspring (Haque *et al*., 2019; El Ahdab *et al*., 2021; Noel *et al*., 2022, 2024). In contrast to the type 1 pro-inflammatory immune responses [e.g. interferon-*γ*, tumour necrosis factor-*α*, CD4 + T helper type 1 (Th1) cells] triggered by bacterial or viral infections, similar to most GI nematodes, infection with *H. bakeri* elicits a type 2 host resistance and tolerizing immune response [e.g. interleukin (IL)-4, IL-5, IL-13, Th2 cells] (Maizels *et al*., 2012; Reynolds *et al*., 2012; Chen *et al*., 2023). *Heligmosomoides bakeri* also induces an immunoregulatory network that aids long-term survival in its host, involving proliferation of Foxp3 + CD4 + regulatory T (Tregs) cells and the potent immunoregulatory cytokines IL-10 and transforming growth factor (TGF-*β*) (Maizels *et al*., 2012; Reynolds *et al*., 2012). This also allows the nematode to dampen inflammatory and pathologic processes, and prevent or ameliorate a number of hyper-immune/inflammatory diseases (Elliott *et al*., 2004; Wilson and Maizels, 2006; Saunders *et al*., 2007; Smallwood *et al*., 2017; White *et al*., 2020).

Brain gene expression in 7-day-old neonatal male offspring born to *H. bakeri*-infected CD-1 outbred dams revealed upregulation of 5 key interacting pathways associated with long-term potentiation (LTP) (Haque *et al*., 2019), the cellular mechanism of learning and memory (Dong *et al*., 2015). This was consistent with gene expression and electrophysiological data indicating enhanced hippocampal LTP in 3-week-old uninfected offspring (Noel *et al*., 2024). The hippocampus is involved in cognitive functions, and also plays an important role in the regulation of emotional behaviours, particularly anxiety (Ghasemi *et al*., 2022). Spatial learning and memory in rodents is critically dependent on hippocampal synaptic plasticity (Clark and Martin, 2005; Dringenberg, 2020); thus, enhanced hippocampal LTP was consistent with the enhanced spatial memory that was observed in 2- and 3-week-old uninfected male and female offspring of *H. bakeri*-infected dams (Noel *et al*., 2022). Furthermore, maternal *H. bakeri* infection resulted in a Th2/Treg biased neuroimmune environment in the uninfected offspring. Gene expression analysis of the brains of neonates of infected dams revealed upregulated Th2/Treg pathways, including the genes for the potent immunoregulatory cytokines IL-4 and TGF-*β*, and downregulated Th1/Th17 pathways (Haque *et al*., 2019; El Ahdab *et al*., 2021), mimicking the immune response of the infected mother (Odiere *et al*., 2013; Su *et al*., 2023). This altered neuroimmune environment was also seen in the hippocampus of male and female 3-week-old juvenile offspring where the immunoregulatory TGF-*β* signalling pathway was upregulated, and where a greater number of 2 immune sensitive cells, microglia and astrocytes, were observed, as well as a higher percentage of CD206-positive microglia (Noel *et al*., 2024) which are typically increased in response to the Th2 cytokine, IL-4 (Francos-Quijorna *et al*., 2016; Liu *et al*., 2016; Jurga *et al*., 2020; Zhang *et al*., 2021). These findings reveal a potential mechanism behind the enhanced LTP and spatial memory of uninfected offspring, as both TGF-*β* and IL-4 are known to have measurable downstream effects on LTP and spatial memory (Nolan *et al*., 2005; Fukushima *et al*., 2007; Derecki *et al*., 2010; Gadani *et al*., 2012; Caraci *et al*., 2015). Whether the improved spatial memory extends to adult offspring is, however, unknown.

The Barnes maze test (BMT) has been previously used to assess spatial learning and memory of 3-week-old offspring in response to maternal *H. bakeri* infection (Noel *et al*., 2022). This test assesses hippocampus-dependent spatial reference memories formed over repeated trials in an unchanging environment (Sharma *et al*., 2010) by measuring the ability of rodents to learn and recall the location of a goal box which is located under 1 of 20 holes around the perimeter of a platform (Sharma *et al*., 2010). It involves a habituation trial (day 0), a training phase (days 1–4) to test spatial learning, both of which contain the goal box, and probe trials 1 (day 5) and 2 (day 12), with no goal box, to test short- and long-term spatial reference memory, respectively. Of note, although the BMT is primarily designed to assess spatial learning and memory, it can also be used to explore 2 indicators of anxiety-like behaviour. First, when rodents are initially placed in an environment that is distinctly different from any environment they have previously encountered, they become afraid or anxious, and defecate (Denenberg, 1969; Bailey and Crawley, 2011). Thus, quantifying the number of fecal pellets during the 5 min habituation trial when mice are first introduced to the BMT can be used as an indicator of their anxiety/fear level, with a greater number of fecal pellets indicating a higher level of anxiety/fear (Denenberg, 1969; Bailey and Crawley, 2011). Second, mice tend to favour darker, more enclosed spaces and thus avoid exploring open areas, especially when they are brightly lit (Bailey and Crawley, 2011). Thus, during the BMT training trials, mice are motivated to seek shelter from the brightly lit, exposed maze, by entering the dark enclosed goal box. It can therefore be interpreted that mice that find the goal box, but choose instead to continue exploring the maze, may be less fearful/anxious than mice who immediately enter the goal box upon finding it.

Using the BMT, the goal of this study was to determine if the positive impact of maternal *H. bakeri* infection during pregnancy and lactation on spatial memory of juvenile offspring was retained in adult offspring. Indicators of anxiety-like behaviour in these offspring were also explored. This study presents the first evidence that enhanced spatial memory previously observed in juvenile offspring in response to a maternal GI nematode infection is retained in uninfected adult female, but not male, offspring, and that these adult females also have reduced anxiety-like behaviour compared to female offspring of uninfected dams.

## Materials and methods

### Experimental design

A 2 × 2 factorial design was employed using *H. bakeri* infected *vs* uninfected dams, and their male *vs* female offspring. All procedures were approved by the McGill University Animal Care Committee according to the guidelines of the Canadian Council on Animal Care.

### Mice and parasites

**Figure 1.**
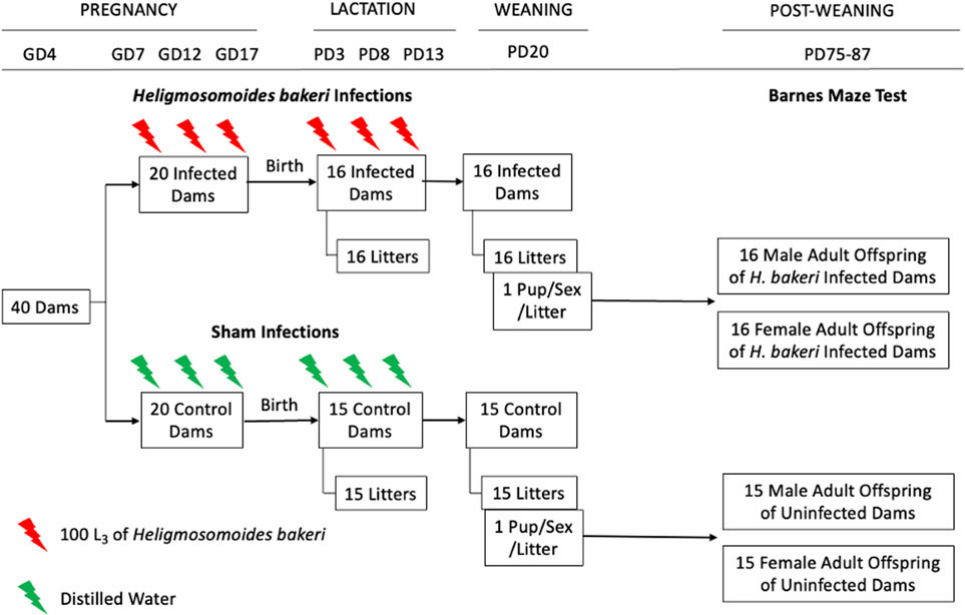
Schematic representation of experimental design and protocol. Of the 40 timed-pregnant dams received on gestation day (GD) 4, only 31 delivered litters. On postnatal day (PD) 20, 1 pup per sex per litter was selected to perform the Barnes maze test. Of the pups selected for behavioural analysis, their size, specifically crown-rump length and weight, were recorded on PD 20 and 69 (see Supplementary Fig. 2).

Of the 40 primiparous 8-week-old timed pregnant outbred CD-1 mice that were received from Charles River Laboratories (Quebec, Canada) on gestation day (GD) 4, 31 were pregnant (78% pregnancy rate). Each dam with her litter was housed individually in a Nalgene cage (Fisher Scientific, Montreal, Canada) at 21–23°C, 40–60% relative humidity and a 12 h light and dark cycle. Mice had *ad libitum* access to a 2920X Teklad rodent diet (18% crude protein, 5% crude fat, 5% crude fibre). Within each of the 6 staggered groups of dams received over 3 months, dams were randomized into uninfected and infected groups, providing a total of 15–16 dams per group were used for this study. This provided an acceptable sample size based on a minimum of at least 6 dams per treatment condition (Meyer *et al*., 2009). Using standard *H. bakeri* protocols (Johnston *et al*., 2015), infective L3 were obtained by fecal culture of stock parasites maintained in outbred CD-1 mice. In our previous studies on the impact of maternal *H. bakeri* infection on uninfected offspring (Haque *et al*., 2019; Noel *et al*., 2022, 2024), a trickle infection protocol was used to maximize antigenic stimulation during pregnancy and lactation and to simulate ongoing natural infection that occurs in wild mice (Brailsford and Behnke, 1992). This same protocol was used here. Dams in the infected group were intubated using an oral gavage needle with 100 ± 3 L3 suspended in 0.1 mL distilled water on GD 7, 12, 17, and postnatal day (PD) 3, 8, and 13 (Fig. 1). Uninfected dams were intubated at the same frequency with 0.1 mL distilled water, to control for any stress due to handling. Given that *H. bakeri* eggs released into the environment develop into infective larvae after 7 days, all cages were cleaned every 5 days to ensure offspring could not ingest infective larvae. Dams were weighed on GD 7, 12, and 17. Following weaning (PD 20), dams were euthanized and necropsied to confirm successful infection of dams based on the presence of adult worms in the small intestine.

Pups were born on GD 19, litter size was recorded on PD 3, 8, 13, and 20, and body mass and length from the top of the head to the base of the tail were recorded on PD 20 and 69. At PD 20, pups were weaned, sexed, and given a unique identifier with a permanent marker, and 1 male and 1 female pup per litter were randomly selected for the BMT (Fig. 1). Pups selected for the adult BMT were housed with 2 littermates of the same sex until testing was performed 2 months later. After the BMT, experimental pups were necropsied and intestines were examined for adult *H. bakeri* to confirm that they had not accidentally become infected. Remaining pups were used for a separate experiment (Noel *et al*., 2024).

### Experimental room

The BMT was conducted in a quiet room (340 cm × 260 cm) which was brightly lit (a floor lamp in each corner and an over-head light) to provide a mild negative reinforcement. Trials were recorded using an overhead monochromatic video camera (Basler Ace monochrome) connected to a computer located in the back corner of the room behind a curtain. The experimenter remained behind the curtain during all recordings. Data were extracted from the videos using the Ethovision XT software (version 17). All equipment remained in the same location in the room, providing visual spatial cues.

### Barnes maze test

The BMT procedure followed a protocol (Sunyer *et al*., 2007) that was previously used to successfully test spatial learning and short- and long-term reference memory in juvenile CD-1 mice born to uninfected or *H. bakeri*-infected dams (Noel *et al*., 2022), as well as adult CD-1 mice born to uninfected dams (Patil *et al*., 2009). As previously described (Noel *et al*., 2022), the Barnes Maze (Maze engineers, 412 Wilmette Ave, Glenview, IL 60025, USA) is an opaque circular platform (diameter: 92 cm, height: 70 cm) with 20 equally spaced holes (diameter: 5 cm) located 2 cm from the edge. In a brightly lit environment, mice naturally seek the dark enclosed area provided by the black goal box (20 × 10 × 4 cm) located under the same escape hole throughout all trials. From the surface of the maze, the escape hole, containing the goal box, looks identical to the other 19 holes. Mice learn the location of the goal box based on spatial cues in the room.

The BMT was conducted when offspring were 3 months old. The BMT involved a habituation trial (day 0), a training phase (days 1–4) to test spatial learning and probe trials 1 (day 5) and 2 (day 12) to test short- and long-term spatial reference memory, respectively. Home cages were moved into the experimental room for 15–20 min acclimation prior to trials and all equipment was cleaned with 70% ethanol between trials.

The habituation trial was used to introduce the mouse to the apparatus and reduce anxiety during the test. The mouse was placed in an opaque starting cylinder (diameter: 10.5 cm, height: 8 cm) at the centre of the platform. After 10 s, the cylinder was removed, and the animal was allowed to freely explore the apparatus for 5 min. After 5 min, the mouse was guided to the goal box and remained there for 2 min. During the habituation trial, the number of fecal droppings was counted, as an indicator of anxiety (Denenberg, 1969).

Training involved four 3 min trials per day for 4 training days. Each of the 16 training trials began by placing the mouse in the starting cylinder at the centre of the platform. After 10 s, the cylinder was removed, recording began and the animal was allowed to freely explore the apparatus for 3 min. Once the animal entered the goal box, it was allowed to remain there for 1 min. Mice that failed to enter the goal box within 3 min were gently guided to its location and placed inside. After each of the four 3 min training trials per day, mice were returned to their home cage for 20 min. During the training trials, the following variables were recorded: (1) total latency (s), defined as time taken until the mouse enters the goal box; (2) total distance (cm), defined as distance travelled until the mouse enters the goal box; (3) total errors, defined as number of times the mouse visited non-escape holes (noted as nose pokes into holes), before entering the goal box; and (4) mean velocity (cm s^−1^) used to determine if performance differences reflected motor ability. If a mouse did not enter the goal box during a 3 min training trial, 180 s was entered as their total latency, and their number of errors and distance travelled during the 3 min trial were entered as total errors and total distance, respectively. As previous studies have observed that mice may find the goal box but choose to continue exploring the maze (Harrison *et al*., 2006; Patil *et al*., 2009), it is recommended to also record latency, distance and number of errors to the first encounter (nose poke) of the escape hole, called primary latency, primary distance and primary errors, respectively, to assess spatial learning. The number of trials where mice did not enter the goal box was also recorded.

Prior to probe trials 1 and 2, the goal box was removed from the escape hole and mice explored the maze for 90 s. No training occurred between the 2 probe trials. Primary latency, primary distance and primary errors were recorded during the probe trials.

### Statistical analyses

Statistical analyses were performed in R statistical software 4.2.3 (R Core Team, 2020), and figures were produced using GraphPad Prism V9. For comparisons over time, where there were repeated measures (i.e. pup size and spatial learning), models were built with maternal treatment condition (*H. bakeri* infected *vs* uninfected), offspring sex (male *vs* female) and timepoint/trial included as a fixed factors and the identity of the mouse as a random factor (Lazic, 2010). A similar model was built for dam weight. Litter size was included as a covariate when assessing mouse size (pup and dam). Further, as body weight has been shown to negatively influence spatial learning (Cordner and Tamashiro, 2015), and we observed this in our study, offspring weight was included as a covariate in our models for spatial learning. No association was found between body weight and spatial memory performance, thus body weight was not included as a covariate in these models. Non-significant interactions between fixed effects were excluded from models (Zuur *et al*., 2009). As trends in the anxiety-like behaviour and spatial memory data were observed, where differences were evident between females born to *H. bakeri* infected *vs* uninfected dams but not between males, data from male and female offspring were analysed separately. This approach is often taken in the literature (Lante *et al*., 2007; Batinić *et al*., 2016); thus, models were built with maternal treatment condition (*H. bakeri* infected *vs* uninfected) as the fixed factor.

Extreme outliers were identified in Prism using the ROUT method (a method combining Robust regression and Outlier removal). The strictest cut off of *Q* = 0.1% was selected to reduce the chance of falsely detecting outliers, meaning only extreme outliers were identified and removed. Extreme outliers occurred only in probe trial 1 where 2 male treatment mice were outliers for primary latency, distance and errors. Analyses for probe 1 were performed with these mice included and excluded and exclusion of these outliers did not influence the results.

Using the fisher.test function in R, Fisher's exact tests were used to analyse the number of trials where mice did or did not enter the goal box during the training phase; male and female offspring were analysed separately. For remaining variables, linear models (LM), negative binomial generalized linear models (NB.GLM), linear mixed models (LMMs) or generalized linear mixed models (GLMMs) were built using the lm, glm.nb, nlme or glmer function, respectively [MASS package (Venables and Ripley, 2002), nlme package (Pinheiro, 2023) and lme4 package (Bates *et al*., 2015)]. When necessary, *post hoc* pairwise comparisons were performed using the emmeans function [emmeans package (Lenth, 2020)] with a Tukey correction. Normality, independence and homogeneity of variances of mixed models were assessed using fitted residuals from the plotresid function [RVAideMemoire package (Hervé, 2020)], and in the case of GLMMs, also using the DHARMa package (Hartig, 2020). Unless otherwise stated, values are presented as means ± s.e.m. The significance level was set at 0.05.

As no pup mortality occurred, the influence of the maternal infection status on litter size was analysed on PD 20 using a LM. For dam weight and offspring weight and length, measured over time, LMMs were used with log transformations for weight and length.

Data on number of fecal droppings were discrete and overdispersed and were analysed using NB.GLMs. Variables from the training and probe trials were positively skewed, and in some instances, heteroscedastic. For the training phase, LMMs with log transformations were used for total latency, total distance and mean velocity and Gamma GLMMs, with log link function, were used for primary latency and primary distance. Both total and primary errors were discrete and overdispersed, and negative binomial GLMMs, with log link function, were used. For the probe trials, LMs with log transformations were used for primary latency and primary distance and a NB.GLM was used for primary error.

## Results

This study assessed the influence of maternal *H. bakeri* infection on the spatial learning and memory and anxiety-like behaviour of uninfected male and female adult offspring in the BMT. Outbred CD-1 mice were infected repeatedly or sham-infected during pregnancy and lactation and their 3-month-old adult offspring from 15 uninfected and 16 *H. bakeri*-infected dams were used (1 male and 1 female pup per dam). Mortality was consistently zero in this infection model, as expected (Noel *et al*., 2022).

### Impact of maternal infection on dam mass, litter size, and pup size

Maternal infection did not influence dam mass at GD 7, 12 or 17 (all *P* values >0.05) or litter size (uninfected: 12.2 ± 0.4; infected: 11.8 ± 0.3; *P* = 0.44) (Supplementary Fig. 1). As reported in the literature (Kristan, 2002; Odiere *et al*., 2010; Noel *et al*., 2022), male and female pups born to infected dams had lower mass and shorter length than pups of uninfected dams at PD 20 (all *P* values <0.001, Supplementary Fig. 2). For the first time, it was observed that this impaired growth persisted in adult offspring at PD 69 (all *P* values <0.001, Supplementary Fig. 2). As expected, mass and length were greater in males than females (Kristan, 2002; Noel *et al*., 2022) at both PD 20 and PD 69 (all *P* values <0.01, Supplementary Fig. 2).

### Impact of maternal infection on offspring anxiety-like behaviour and spatial learning and memory in the Barnes maze test

#### Anxiety-like behaviour during habituation trial

**Figure 2.**
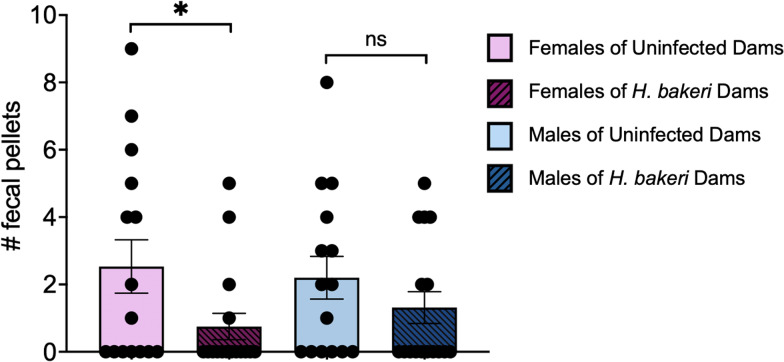
Maternal *H. bakeri* infection reduced female offspring fecal count during the 5 min habituation trial of the Barnes maze test. Values are means±s.e.m., *n* = 15–16 offspring per group (*P < 0.05; ns = not significant).

Defecation is a sign of fear or anxiety in rodents, which is often observed when they are placed in a novel environment (Denenberg, 1969; Bailey and Crawley, 2011). Thus, the number of fecal pellets was counted during the 5 min habituation trial when mice were introduced to the maze, as a measure of anxiety-like behaviour. Female offspring of *H. bakeri*-infected mothers had significantly fewer fecal pellets than female offspring of uninfected mothers (*P* = 0.02; Fig. 2), suggesting that maternal infection during pregnancy and lactation reduced anxiety in their uninfected adult female offspring. This difference was not observed in the males.

#### Spatial learning and anxiety-like behaviour in the training phase

As mice may continue to explore the maze after finding the goal box, 2 sets of spatial learning variables were explored, those related to first arriving at the escape hole which contained the goal box (‘primary’ variables), and those related to entering the goal box (‘total’ variables). Primary variables were a better indication of spatial learning whereas total variables were used to assess spatial exploration as an indicator of anxiety-like behaviour.

**Figure 3.**
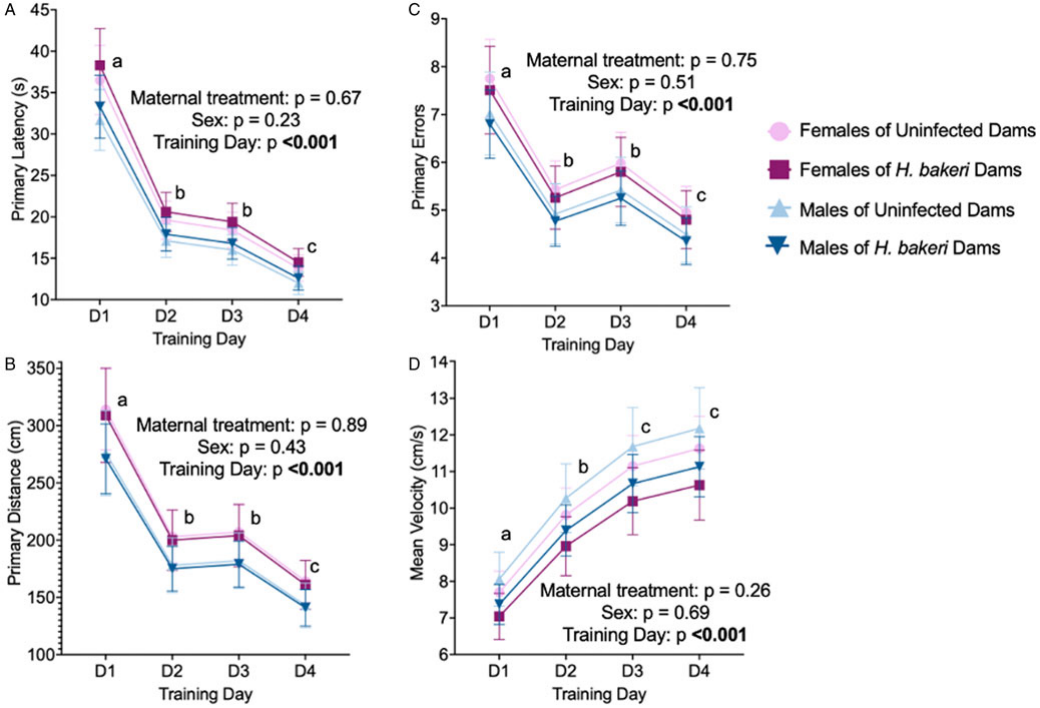
Neither maternal *H. bakeri* infection nor adult offspring sex influenced primary variables of spatial learning in the Barnes maze test over 4 training days. All mice performed significantly better after the first training day. Values are LSmeans±s.e.m., *n* = 15–16 offspring per group. (a) Primary latency, (b) primary distance and (c) number of primary errors to reach the escape hole, and (d) mean velocity during the trial. Different letters show the effect of training day, *P* < 0.05.

*Spatial learning*: Regardless of maternal infection or offspring sex, adult offspring learned the location of the escape hole, based on the first nose poke into the escape hole, on the first training day as indicated by a decrease in the average primary latency (*P* < 0.0001; Fig. 3a), primary distance (*P* < 0.0001; Fig. 3b) and primary errors (*P* < 0.0001; Fig. 3c) between training days 1 and 2. Thereafter, values remained low. Neither maternal infection nor offspring sex influenced mean velocity (Fig. 3d), indicating no differences in motor ability. Of note, independent of maternal infection, primary latency and primary distance were negatively associated with offspring weight (all *P* values < 0.05, data not shown).

**Figure 4.**
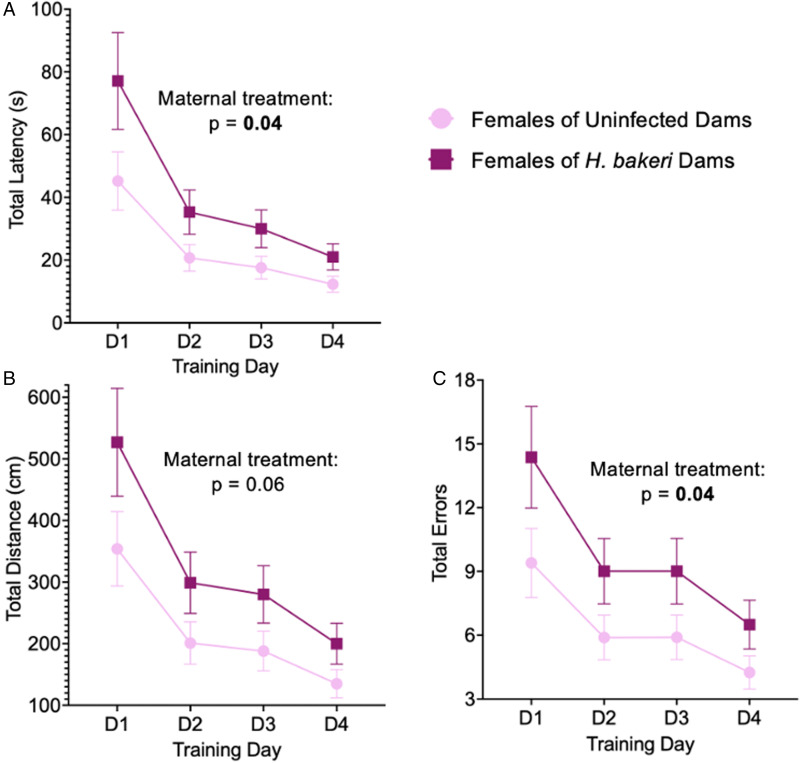
Maternal *H. bakeri* infection influenced female offspring exploration during the 4-day training phase of the Barnes maze test. Total parameters were used as an indication of exploration to provide an understanding of fear/anxiety levels. Values are LSmeans±s.e.m., *n* = 15–16 offspring per group. (a) Total latency, (b) total distance and (c) number of total errors to enter the goal box.

*Anxiety-like behaviour*: Adult female offspring of infected mothers had higher total latency (*P* = 0.044; Fig. 4a), total distance (*P* = 0.058; Fig. 4b) and total errors (*P* = 0.043; Fig. 4c) compared to female offspring of uninfected mothers. No differences were observed between adult male offspring of uninfected and infected mothers in total latency, total distance and total errors (Supplementary Fig. 3). Furthermore, although all mice found the goal box during each training trial, a significantly higher percentage of females of infected dams did not enter the goal box (17.2%) compared with females of uninfected dams (4.6%) (*P* < 0.0001). There was no difference in the percentage of training trials where males did not enter the goal box (males of infected mothers: 6.6%; males of uninfected mothers: 4.6%; *P* = 0.34). Taken together, these data indicate that females of infected mothers were less anxious as they were less motivated to seek shelter in the goal box during training trials, and more inclined to explore the maze after finding the goal box.

#### Probe trials of spatial memory

**Figure 5.**
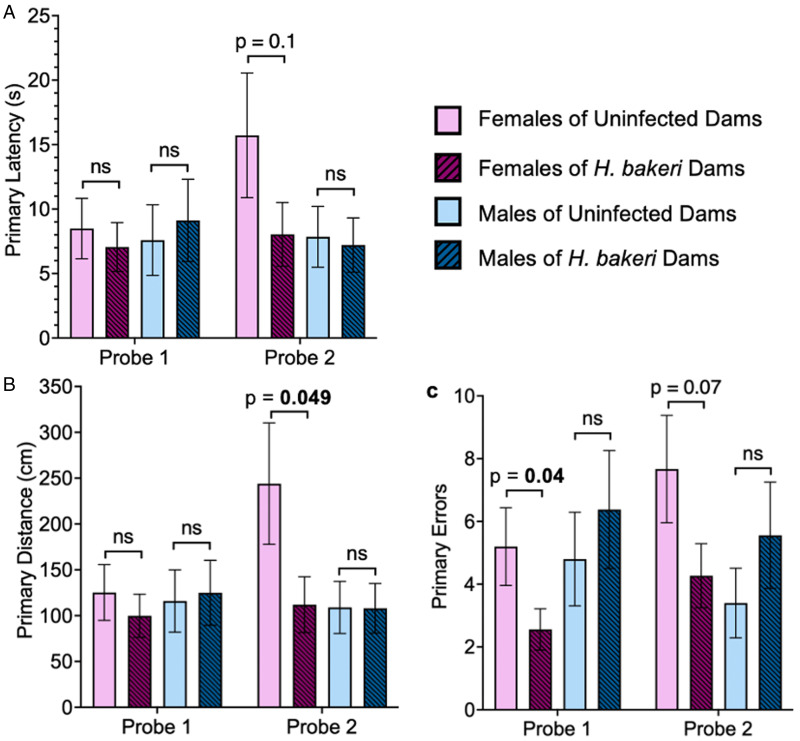
Maternal *H. bakeri* infection influenced adult female offspring short-term (probe trial 1) and long-term (probe trial 2) spatial reference memory in the Barnes maze test but not male offspring spatial reference memory. Probe trial 1 was conducted 24 h after the last training day and probe trial 2 was conducted 1-week later. Values are means±s.e.m., *n* = 14–16 offspring per group (ns = not significant). (a) Primary latency, (b) primary distance and (c) number of primary errors to reach the escape hole.

During probe trial 1, which assessed short-term spatial reference memory, maternal infection did not influence the time taken (primary latency) or distance travelled (primary distance) to find the escape hole for adult female offspring (Fig. 5a–b). However, adult female offspring of infected mothers made half as many primary errors before first finding the escape hole compared to offspring of uninfected mothers (*P* = 0.043; Fig. 5c) indicating better short-term spatial reference memory in response to maternal *H. bakeri* infection. These differences were not detected in the adult male offspring (Fig. 5a–c).

During probe trial 2, which assessed long-term spatial reference memory, females of infected mothers travelled a shorter primary distance (*P* = 0.049; Fig. 5b) and although not significant, they appeared to make fewer primary errors (*P* = 0.073; Fig. 5c) in finding the escape hole compared to females of uninfected mothers. These results provide evidence of better long-term spatial reference memory in adult female offspring of infected dams. There were no differences in long-term spatial reference memory between males of uninfected or infected mothers (all *P* values in probe 1 and 2 > 0.05; Fig. 5a–c). The fewer primary errors in probe trial 1 and shorter primary distance in probe trial 2 provide evidence that maternal infection may enhance spatial memory in adult female mice.

## Discussion

Using a GI nematode parasite that remains in the maternal intestine, the main goal of this study was to assess if the positive influence of maternal infection during pregnancy and lactation on spatial memory of juvenile male and female offspring was retained in the uninfected adult offspring. Some indicators of anxiety-like behaviour in adult offspring were also explored. It was reported for the first time that female adult offspring of *H. bakeri*-infected dams retained enhanced spatial reference memory and also exhibited signs of reduced anxiety-like behaviour compared to females of uninfected dams. No differences were observed in the behaviour of adult male offspring of infected *vs* uninfected dams. It is hypothesized that sex hormones may at least in part explain the sex-specific differences in behavioural responses to maternal infection of adult offspring.

Previous findings showed that maternal *H. bakeri* infection enhanced spatial memory in both juvenile male and female offspring, where PD 17 pups of infected dams retained object location memories for 3 h in the object location test but offspring of uninfected mothers did not, and where PD 34 juveniles of infected mothers retained their ability to find an escape location in the BMT for 1 week but offspring from uninfected mothers did not (Noel *et al*., 2022). These findings were consistent with the enhanced hippocampal LTP in uninfected 3-week-old offspring of *H. bakeri*-infected dams as evidenced by both gene expression and electrophysiological data, and the upregulated neurogenesis pathway (Noel *et al*., 2024), all of which are strongly associated with spatial memory (Abrous and Wojtowicz, 2015; Lieberwirth *et al*., 2016). Further, maternal *H. bakeri* infection also upregulated the expression of brain-derived neurotrophic factor (BDNF) in the whole brain of neonates (unpublished data from our lab; *P* = 6.8 × 10^−5^), and hippocampus of juveniles (Noel *et al*., 2024). BDNF is a key molecule for learning and memory as it is involved in neurogenesis and synaptic plasticity (Lu *et al*., 2008; Miranda *et al*., 2019), and it also plays an important role in reducing behaviours associated with anxiety (Tatiana Marins *et al*., 2020; Yin *et al*., 2022). It has been suggested that these differences may be due to the Th2/Treg biased neuroimmune environment observed in the hippocampus of uninfected male and female juvenile offspring of *H. bakeri*-infected mothers (Noel *et al*., 2024), a neuroimmune environment that mimics the systemic immune response of the infected mother (Odiere *et al*., 2013; Su *et al*., 2023).

The present study has shown that the enhanced spatial reference memory observed in juvenile offspring in response to maternal *H. bakeri* infection (Noel *et al*., 2022) was retained in adult female offspring. This was evidenced by adult female offspring of infected mothers making half the number of errors before finding the escape hole in both probe trials, and having a more direct path to find the escape hole in probe trial 2 in comparison to adult females of uninfected mothers. These differences were not observed between male offspring of infected *vs* uninfected mothers. The present study also provides evidence of decreased anxiety-like behaviour in adult female offspring of *H. bakeri*-infected mothers, as they produced fewer fecal pellets in the 5 min habituation trial, compared with the adult female offspring of uninfected mothers. In addition, during the training phase, despite finding the goal box which offered protection from the brightly lit and exposed maze, adult female offspring of infected dams chose to continue exploring instead of seeking shelter and thus did not enter the goal box as soon as females of uninfected mothers. Again, these differences were not observed between male offspring of infected *vs* uninfected mothers. Previous studies have found that maternal physical exercise enhances offspring spatial memory and decreases anxiety-like behaviour in 3-week-old juveniles and 4-month-old adult rats of both sexes (Aksu *et al*., 2012; Dayi *et al*., 2012) and that this is associated with an increase in hippocampal BDNF and neurogenesis (Bick-Sander *et al*., 2006; Lee *et al*., 2006; Kim *et al*., 2007; Aksu *et al*., 2012; Dayi *et al*., 2012; Robinson and Bucci, 2012; Akhavan *et al*., 2013; Gomes da Silva *et al*., 2016). Considering that juvenile male and female offspring of infected mothers have enhanced hippocampal LTP and upregulated genes associated with BDNF and neurogenesis (Noel *et al*., 2024), it is hypothesized that heightened hippocampal BDNF, neurogenesis and LTP may persist in adult females of *H. bakeri*-infected dams, driving at least in part the enhanced spatial memory and decreased anxiety-like behaviour that was observed.

It is of considerable interest that the enhanced spatial memory in response to maternal *H. bakeri* infection persisted into adulthood in female but not male offspring. Previous maternal infection models, which have primarily used bacterial or viral infections, or their mimics, have observed sex-dependent effects of maternal infection on the behaviour of mouse offspring (Bergdolt and Dunaevsky, 2019). Unlike an *H. bakeri* infection which regulates the host immune system, limits pathology and can be considered ‘anti-inflammatory’ in nature (Maizels *et al*., 2012; Reynolds *et al*., 2012), bacterial and viral infections are associated with inflammation and harmful pathology in their host (Britton and Saunders, 2010; Rouse and Sehrawat, 2010). In these maternal bacterial and viral models, the strong proinflammatory Th1 immune response in the infected mother is mimicked in the offspring brain and is associated with impaired hippocampal LTP and spatial memory (Boksa, 2010; Bergdolt and Dunaevsky, 2019). In response to maternal LPS or PolyI:C, male offspring display significant spatial learning and memory impairments, whereas females do not (Lante *et al*., 2007; Howland *et al*., 2012; Wischhof *et al*., 2015; Batinić *et al*., 2016; Gogos *et al*., 2020). The ability of the female sex hormones, oestrogen and progesterone, to dampen Th1 immunity (Roved *et al*., 2017) may offer protection to female offspring in these models, by ameliorating the damaging neuroinflammatory responses. In contrast to LPS and Polyl:C models, maternal *H. bakeri* infection induces a Th2/Treg, not a Th1, immune response in the mother (Odiere *et al*., 2013; Su *et al*., 2023), which is mimicked in the brain of neonatal and juvenile offspring and is suggested to promote hippocampal BDNF, neurogenesis, LTP and spatial memory (Haque *et al*., 2019; El Ahdab *et al*., 2021; Noel *et al*., 2024). Given that oestrogen and progesterone enhance Th2 immune responses (Roved *et al*., 2017), it is possible that the influence of these sex hormones on the immune system may allow the Th2/Treg biased neuroimmune environment to persist in adult female offspring of *H. bakeri* dams, explaining the positive influence on female behaviour at adulthood.

In contrast to oestrogen and progesterone, the male sex hormone, testosterone, dampens Th2 immune responses (Hepworth *et al*., 2010; Klein and Flanagan, 2016; Roved *et al*., 2017; Taneja, 2018). Evidence of this is seen in mice infected with *H. bakeri* (Dobson, 1961; Van Zandt *et al*., 1973; Prowse *et al*., 1979; Maizels *et al*., 2012; Rynkiewicz *et al*., 2019) or the GI nematode *Trichuris muris* (Hepworth *et al*., 2010), where male mice are less resistant and harbour more worms than female mice (Dobson, 1961; Van Zandt *et al*., 1973; Prowse *et al*., 1979; Maizels *et al*., 2012; Rynkiewicz *et al*., 2019), due to the suppression of protective Th2 immunity by testosterone in males (Hepworth *et al*., 2010; Roved *et al*., 2017). It is thus possible that the Th2/Treg biased neuroimmune environment observed in juvenile male and female offspring of *H. bakeri*-infected dams (Noel *et al*., 2024), which was hypothesized to promote spatial memory (Noel *et al*., 2022, 2024), may be retained in adult female offspring but dampened by testosterone in adult males, explaining why enhanced spatial memory in adult male offspring was no longer observed. Given that circulating sex hormones are at low levels in prepubescent mice (Bell, 2018), they would not have been expected to have a strong influence on the immune response in juvenile offspring.

The ecological effects of a GI nematode infection that enhances spatial memory and reduces anxiety-like behaviour of female offspring are unknown. Spatial memory in mice is necessary for mate location, foraging, predator avoidance and territorial defence, and it is therefore an essential aspect of survival (Vorhees and Williams, 2014). Enhancement of spatial memory might thus be beneficial. Additionally, the reduced anxiety-like behaviour might complement the enhanced spatial memory, as adult female offspring of *H. bakeri*-infected dams would be less anxious and more inclined to explore, increasing opportunities for foraging. Of note, however, reduced anxiety-like behaviour could be a disadvantage with respect to safety and predator avoidance. Lastly, given that reproductive effort and parental investment are more costly for female mice than for males (Parmigiani *et al*., 1994), there could be evolutionary advantages if females have better spatial memory and lower anxiety.

The following limitations are acknowledged. First, the oestrous cycle of adult female offspring was not controlled for. Although oestrous cycle is recognized as a strong determinant in emotionality and cognitive capacity of female rodents (Gawel *et al*., 2019), the large sample size, and use of the BMT which spanned 13 days [i.e. 3 oestrous cycles/female mouse (Byers *et al*., 2012)], makes it likely that all the phases were represented. Second, a test designed to specifically assess anxiety behaviour in mice (e.g. elevated plus maze) would have provided more conclusive data on the influence of maternal *H. bakeri* infection on anxiety behaviour in offspring. Third, we were unable to determine whether the neuroimmune environment of adult female offspring of infected dams was altered in response to maternal *H. bakeri* infection, as it had been in the juvenile offspring of infected dams (Noel *et al*., 2024).

To the best of our knowledge, this is the first study to assess the impact of a maternal GI helminth infection on the spatial memory of adult offspring and to determine if the positive influence observed in juvenile male and female offspring persisted into adulthood. It was observed that maternal GI nematode infection during pregnancy and lactation enhanced spatial memory and may also reduce anxiety-like behaviour in adult female, but not male, offspring. It will be of great interest to determine whether sex hormones are the driving factor behind these observations, if this maternal infection influences other aspects of offspring behaviour, and if other nematode infections, such as *Nippostrongylus brasiliensis*, also alter offspring behaviour.

## Supporting information

Noel et al. supplementary material Noel et al. supplementary material

## Data Availability

The datasets generated and analysed for this study are available via a link to the Borealis Dataverse [https://doi.org/10.5683/SP3/HVCHM4], a public data repository.

## Supplementary material

The supplementary material for this article can be found at https://doi.org/10.1017/S0031182024000696.
