# Supplementary material for: Gastrointestinal nematode infection during pregnancy and lactation enhances spatial reference memory and reduces indicators of anxiety-like behaviour in uninfected adult female mouse offspring
Source: Parasitology. 2024 May 29;151(7):722–31. doi: 10.1017/S0031182024000696 (PMC11474017; doi:10.1017/S0031182024000696)

Supplementary Figure 1. Maternal *H. bakeri* infection did not influence dam weight at gestation day (GD) 7, 12 and 17. Litter size was included as a covariate. Values are means$\pm$SEM, n = 15-16 per group (ns = not significant).


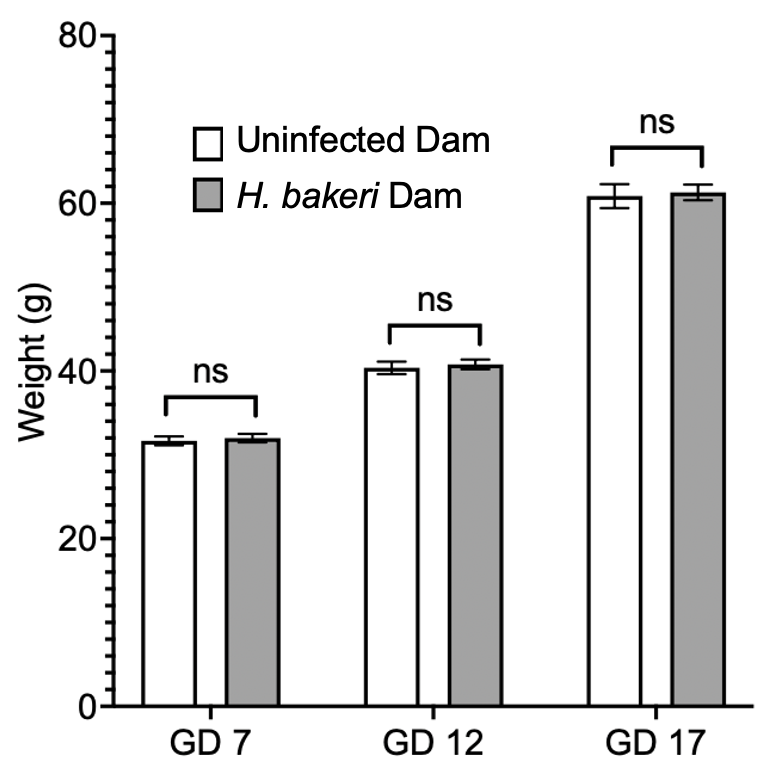


Supplementary Figure 2. Maternal *H. bakeri* infection influenced offspring size at postnatal day (PD) 20 and 69. Litter size was included as a covariate. Values are means$\pm$SEM, n = 15-16 offspring per group (****P* < 0.001). **(a)** Offspring body mass and **(b)** offspring crown-rump length.


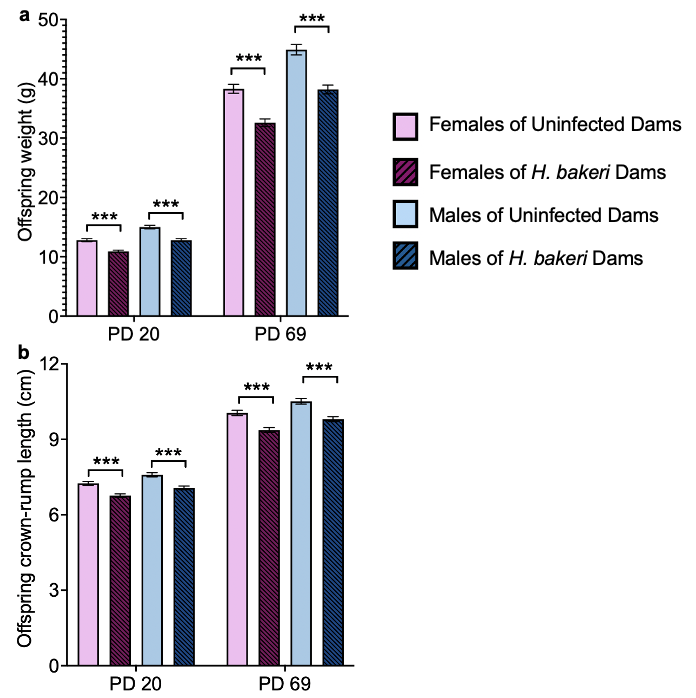


Supplementary Figure 3. Maternal *H. bakeri* infection did not influence male offspring exploration during the four day training phase of the Barnes Maze Test. Total parameters were used as an indication of exploration to provide an understanding of fear/anxiety levels. Values are LSmeans$\pm$SEM, n = 15-16 offspring per group. **(a)** total latency, **(b)** total distance and **(c)** number of total errors to enter the goal box.


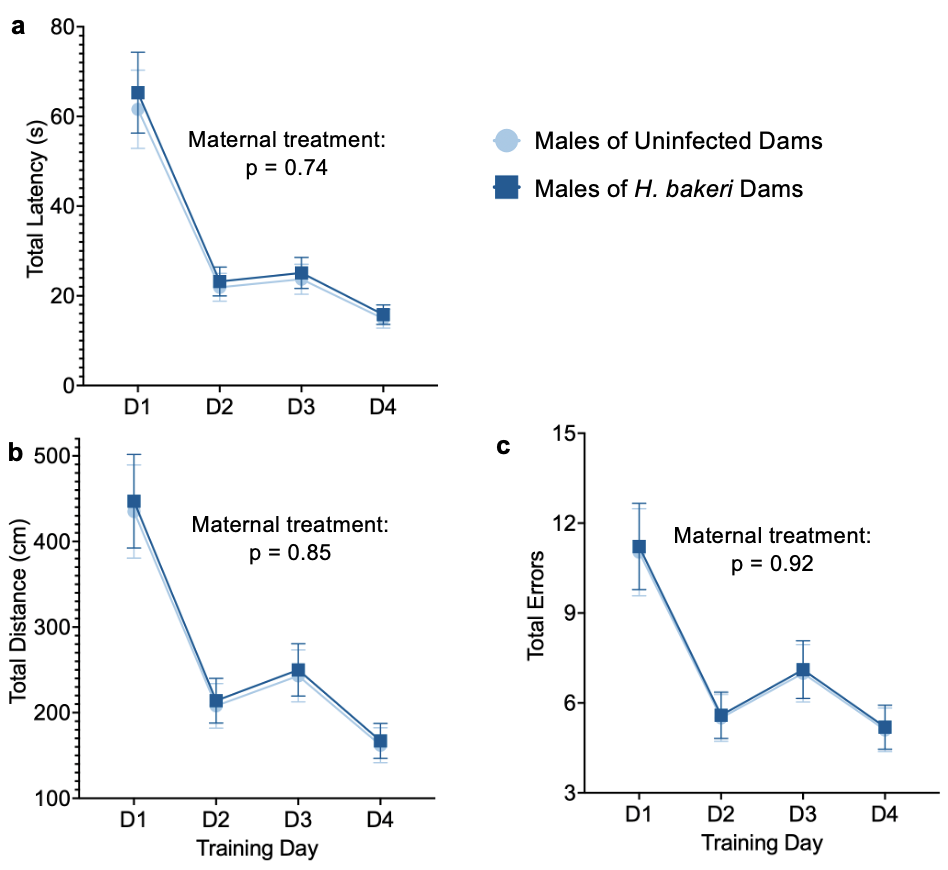

Supplement: Noel et al. supplementary material [file S0031182024000696sup001.docx]
